# Orientation of Polylactic Acid–Chitin Nanocomposite Films via Combined Calendering and Uniaxial Drawing: Effect on Structure, Mechanical, and Thermal Properties

**DOI:** 10.3390/nano11123308

**Published:** 2021-12-06

**Authors:** Shikha Singh, Mitul Kumar Patel, Shiyu Geng, Anita Teleman, Natalia Herrera, Daniel Schwendemann, Maria Lluisa Maspoch, Kristiina Oksman

**Affiliations:** 1 Division of Materials Science, Department of Engineering Sciences and Mathematics, Luleå University of Technology, SE-971 87 Luleå, Sweden; sikha.msc@gmail.com (S.S.); mitul.kumar.patel@ltu.se (M.K.P.); shiyu.geng@ltu.se (S.G.); Natalia.herrera-vargas@storaenso.com (N.H.); daniel.schwendemann@ost.ch (D.S.); 2 Centre Català del Plàstic (CCP)—Universitat Politècnica de Catalunya Barcelona Tech (EEBE-UPC)-ePLASCOM, 08019 Barcelona, Spain; maria.lluisa.maspoch@upc.edu; 3 RISE (Research Institutes of Sweden), SE-114 28 Stockholm, Sweden; Anita.teleman@ri.se; 4 OST Eastern Switzerland University of Applied Sciences, CH-8640 Rapperswil, Switzerland; 5 Mechanical & Industrial Engineering, University of Toronto, Toronto, ON M5S 3BS, Canada; 6 Wallenberg Wood Science Center (WWSC), Luleå University of Technology, SE-971 87 Luleå, Sweden

**Keywords:** PLA, chitin nanocrystals, nanocomposites, extrusion, compression molding, directional orientation, X-ray, mechanical properties

## Abstract

The orientation of polymer composites is one way to increase the mechanical properties of the material in a desired direction. In this study, the aim was to orient chitin nanocrystal (ChNC)-reinforced poly(lactic acid) (PLA) nanocomposites by combining two techniques: calendering and solid-state drawing. The effect of orientation on thermal properties, crystallinity, degree of orientation, mechanical properties and microstructure was studied. The orientation affected the thermal and structural behavior of the nanocomposites. The degree of crystallinity increased from 8% for the isotropic compression-molded films to 53% for the nanocomposites drawn with the highest draw ratio. The wide-angle X-ray scattering results confirmed an orientation factor of 0.9 for the solid-state drawn nanocomposites. The mechanical properties of the oriented nanocomposite films were significantly improved by the orientation, and the pre-orientation achieved by film calendering showed very positive effects on solid-state drawn nanocomposites: The highest mechanical properties were achieved for pre-oriented nanocomposites. The stiffness increased from 2.3 to 4 GPa, the strength from 37 to 170 MPa, the elongation at break from 3 to 75%, and the work of fracture from 1 to 96 MJ/m^3^. This study demonstrates that the pre-orientation has positive effect on the orientation of the nanocomposites structure and that it is an extremely efficient means to produce films with high strength and toughness.

## 1. Introduction

There is a growing worldwide interest in developing bio-based materials for sustainable development to mitigate the waste disposal problems created by petroleum-based polymeric materials. In this context, poly(lactic acid) (PLA), which is derived from natural resources (such as corn and sugar), has gained considerable attention because of its potential properties that are comparable to those of some petroleum-based polymers used for packaging applications [1]. PLA is superior to many petroleum-based polymers because of its bio-compostability, low energy consumption, and low CO_2_ emission. The easy production of PLA at a large scale with a reasonable price expands its applications in many areas, such as packaging, medical, electronic, and fiber materials [2]. However, PLA has some limitations, such as inherent brittleness, poor elongation at break (2–5%), and very low toughness (1 MJ/m^3^), that make its practical use in packaging applications difficult [3]. PLA is brittle for the following reasons: (1) low glass transition temperature (Tg ≈ 55 °C), which makes the polymer chain rigid and inflexible, and (2) slow crystal nucleation, which leads to a large spherulite size [4]. To overcome these limitations, researchers have used plasticizers [5], copolymers [6], and nucleating agents [7] to improve PLA crystallization behavior. However, it has always been challenging to simultaneously fine-tune both tensile strength and toughness. For example, by adding plasticizers to the PLA matrix, the elongation at break increases but the tensile strength and modulus decrease [8].

The orientation of polymer films or tapes significantly improves the properties of polymers, particularly properties such as tensile strength and toughness [9,10]. This orientation can be achieved by two methods: (1) melt-state drawing [9] and (2) solid-state drawing (SSD) [10]. The orientation of the polymer can be affected by processing factors such as the drawing speed, drawing temperature, and draw ratio. Some studies have been published on the effect of orientation produced by drawing PLA films [9,10,11,12,13], most of which have focused on the effect of orientation on crystallization behavior. Mai et al. [10] studied the influence of solid-state orientation on the morphology, mechanical properties, and hydrolytic degradation of extruded PLA tapes. They found that the mechanical properties are dependent on the draw ratio and drawing temperature and that the elongation to break and toughness of the drawn tapes were substantially improved compared to those of the extruded tapes and that the hydrolytic degradation was reduced with increased orientation. Velazquez-Infante et al. [11] studied thermal and mechanical properties of PLA films that were unidirectionally drawn at 22 °C at a speed of 100 mm/min and draw ratio of 4, and they showed that the orientation markedly increased the tensile strength.

While many of these studies have been conducted on neat PLA polymer films, the orientation of PLA nanocomposites has been investigated less. In previous studies from our research group, the uniaxial orientation of PLA–nanocellulose nanocomposites was performed using solid-state and melt-state drawing [14,15,16,17].

Singh et al. studied the effect of drawing conditions (drawing temperature, drawing speed, and draw ratio) on the microstructure, mechanical properties, and thermal properties of PLA–CNF (cellulose nanofiber) nanocomposites. It was found that the toughness of the drawn nanocomposite was 60 times higher than that of undrawn ones. In addition, the orientation also increased the thermal properties and degree of crystallinity [14].

Geng et al. further increased the draw ratio of the PLA–CNF nanocomposites to 8, and showed superior mechanical properties: the tensile strength increased from 64 to 343 MPa, and the toughness increased from 2 to 83 MJ/m^3^, when compared to the un-oriented nanocomposites [15].

The same group showed also that these oriented nanocomposites have great potential in structural applications as well as optical sensors because of their interesting strain-responsive birefringence behavior [16].

Furthermore, a previous study on the melt spinning of PLA nanocomposite fibers reinforced with modified and unmodified cellulose nanocrystals (CNCs) showed that drawing in the melt stage can greatly affect the mechanical properties and that the addition of CNCs (1 wt.%), particularly surface-modified ones, had a significant effect: the modulus improved from 3.4 to 4.1 GPa, the strength increased from 82 to 171 MPa, and the strain increased from 5% to 92% for the nanocomposites [17].

Singh et al. studied solid-state orientation of plasticized PLA-chitin nanocomposites with different ChNC contents (0, 1, 5 wt%) and draw ratios of up to 3 [18]. The results showed that the mechanical properties (strength and stiffness) increased with increasing draw ratio for all materials, but the nanocomposite with a ChNC content of 5 wt% showed superior properties compared to those of the nanocomposites with lower ChNC concentrations. These results confirmed that it is not only the PLA molecular orientation that is the reason for the improved mechanical properties, and this behavior was attributed to the synergistic effect of the orientation of the ChNCs together with the molecular orientation of PLA and crystal growth.

For these reasons, the orientation of nanocomposite with 5 wt% ChNCs was further studied in this work. The hypothesis was formed regarding the possibility of reaching a higher orientation factor if the material is pre-oriented in the melt stage and how this orientation will affect the properties. Therefore, film extrusion followed by calendering was used to pre-orient the nanocomposites before the solid-state drawing (SSD). The pre-orientation was performed by controlling and adjusting the speed of the calendering rolls. These pre-oriented films were then subjected to SSD for further orientation and their orientation index factor was evaluated. The novelty of this work is the combination of these two orientation techniques and the evaluation of the orientation and not only the orientation of the polymer but also the orientation of the ChNCs.

The objective of this study was to achieve highly oriented nanocomposite films by combining film calendaring followed by SSD in uniaxial testing equipment in a temperature-controlled chamber. The oriented nanocomposite films were compared to undrawn compression-molded composite films. The effect of the orientation induced by the presence of ChNCs and the increased degree of orientation on the properties and structure of the nanocomposites were studied using wide-angle X-ray analysis, microscopy, thermal, and mechanical testing.

## 2. Materials and Methods

### 2.1. Materials

Polylactic acid (PLA) was kindly provided by FUTERRO (Escanaffles, Belgium), and used as a polymer matrix. The PLA had MFI of 8 g/10 min (measured at 190 °C and 2.16 kg).

Glycerol triacetate (GTA) was supplied by Sigma-Aldrich (Stockholm, Sweden) and utilized as a plasticizer for the PLA matrix, as well as a dispersion agent, for the ChNCs in the liquid suspension.

The raw chitin powder was provided by Antarctic Seafood SA (Coquimbo, Chile), the detailed description of the isolation process of the chitin nanocrystals (ChNCs) is presented elsewhere [19]. Briefly, the raw chitin was hydrolyzed with 3 M HCl at 100 ± 5 °C under stirring for 90 min. Then, the suspension was diluted with distilled water and washed via centrifugation and transferred to dialysis membranes for 3 days. Before use the suspension was ultrasonicated for 10 min and vacuum filtered using a polyamide filter Sartolon type 250, 0.2 µm pore size (WVR International AB, Stockholm Sweden) to obtain a ChNC gel with a solid content of 19.5 wt%. Ethanol (99.5%) was purchased from Solveco (Stockholm, Sweden).

### 2.2. Methods

The nanocomposite was manufactured using liquid-assisted extrusion process using a co-rotating twin-screw extruder ZSK-25 Coperion W&P (Stuttgart, Germany), the process is explained in detail in previous publications [20,21]. The compositions of the polymer, plasticizer, and ChNCs were in the following order: 75 wt%: 20 wt%: 5 wt%. The temperature profile was ranging from 170 to 190 °C and the material was processed at a screw speed of 250 rpm with a throughput of 15 kg/h. Atmospheric and vacuum venting systems were used to remove liquid phase (steam) during the compounding process.

To prepare undrawn nanocomposite films, extruded PLA–GTA–ChNC pellets were compression-molded in an LPC-300 Fontijne Grotnes press (Vlaardingen, The Netherlands). Approximately 5 g of material was placed between two metallic sheets, heated to 190 °C, and maintained at this temperature for 2 min in contact mode. Then, it was compression molded for 1 min under a pressure of 2 MPa, and rapidly cooled to room temperature using a water-cooling system to make the isotropic nanocomposite films with a thickness approx. 160–180 μm.

**Figure 1:**
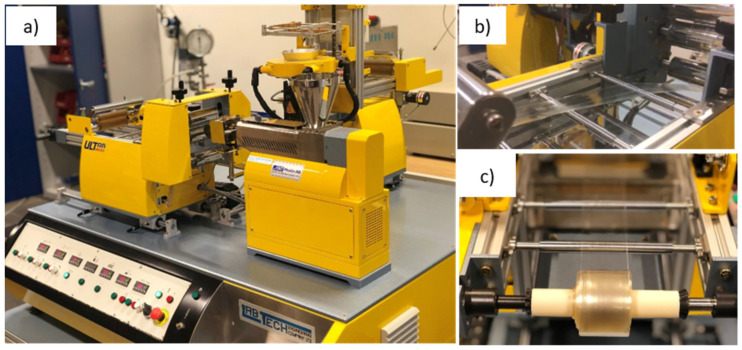
Melt-state drawing process: (**a**) film calendering set-up; (**b**) pressing, stretching, and calendering; and (**c**) film winding process.

The film calendering of PLA–GTA–ChNC nanocomposite was made using a single-screw extruder with an L/D screw ratio of 30:1 (Lab Tech Engineering Company, Ltd., Samutprakan, Thailand). Photographs of the different steps of the FC process are shown in Figure 1. The molten polymer was extruded through a 100 mm slit die at a screw speed of 65 rpm. The extruded films were quenched on a casting roll, accompanied by post drawing on heated rollers at 60 °C (Lab Tech Engineering Company, Ltd., Thailand, type LUMCR-50) to produce the pre-oriented films. The calendered films had thickness between 70–140 μm.

Two processes were used to orient the nanocomposite films: (1) solid-state drawing of compression molded (CM) films and (2) solid-state drawing of pre-oriented films prepared with film calendering (FC). Schematic of the pre-orientation and orientation of the PLA–GTA–ChNC nanocomposites is shown in Figure S1 in Supplementary Information. The nanocomposites films were prepared in two ways in the film calendaring extrusion. First, a similar speed for the stack rolls and wind-up unit was used (no tension applied); the film produced using these parameters is denoted as FC. Second, the nanocomposite was drawn by applying tension between the stripper and haul-off rolls by increasing the speed (2×) of the wind-up unit; the film produced using these parameters is denoted as FC-2. The draw ratio (DR) is defined as the ratio of the drawn film length to the original film length produced in a specified period. The process settings for FC of the nanocomposite films are shown in Table 1.

The solid-state-orientation of films was made by uniaxial drawing using a Shimadzu Autograph AG-X universal testing machine (Kyoto, Japan) equipped with a temperature chamber and a load cell of 5 kN. The orientation of the compression-molded and film-calendered pre-oriented nanocomposite films was conducted at a temperature of 60 °C and a speed of 100 mm/min as described in our earlier study [14]. A gauge length of 10 mm and a sample size of 40 × 6 × 0.1 mm^3^ was used for the drawing. Before drawing, the sample was marked to calculate the DR using Equation (1).

(1) $$\mathrm{Draw}\;\mathrm{ratio}\;\left(\mathrm{DR}\right)=\frac{\mathrm{Final}\;\mathrm{length}\;\mathrm{of}\;\mathrm{the}\;\mathrm{ink}\;\mathrm{mark}\;\left(l\right)}{\mathrm{Original}\;\mathrm{lenght}\;\mathrm{of}\;\mathrm{the}\;\mathrm{ink}\;\mathrm{mark}\;\left(\mathrm{lo}\right)}$$

A DR of 4 was achieved for the CM and FC nanocomposite films. However, this was not possible for the pre-oriented nanocomposite FC-2; for this, the maximum DR was 2.5; however, the total DR for this material was 5, including that from FC (2) and SSD (2.5). Finally, depending on the process used for the orientation and achieved DR, the materials were coded, as shown in Table 2. Nanocomposites processed with compression molding and film calendering are referred to as CM and FC, respectively.

### 2.3. Characterization

X-ray analyses were performed to better understand how the drawing processes contributed to the crystal structure and orientation of the PLA–ChNC nanocomposites. One-dimensional (1D) X-ray diffraction (XRD) patterns were collected using a PANalytical Empyrean X-ray diffractometer (Malvern, UK) with Cu Kα radiation (wavelength 0.15418 nm), in a 2θ angular range of 5° to 40° with a scan rate of 0.01°/s. Two-dimensional (2D) WAXS diffractograms were recorded on an SAXSpoint 2.0 (Anton Paar, Graz, Austria) equipped with a Microsource X-ray source (Cu Kα radiation, wavelength 0.15418 nm) and a Dectris 2D CMOS Eiger R 1M detector with a 75 × 75 µm^2^ pixel size. All measurements were performed with a beam diameter of approximately 500 µm and a beam path pressure of approximately 1 to 2 mbar. The sample-to-detector distance was 111 mm during the measurements. Six frames with a duration of 5 min were read from the detector, giving a total measurement time of 0.5 h per sample. The transmittance was determined and used for scaling the intensities. The orientation indices (fc) of the PLA and chitin nanocrystals were calculated according to the intensity distributions of the azimuthal angle using Equation (2) [22]:

fc = (180° − FWHM)/180° (2)

where FWHM is the full width at half maximum of the azimuthal angle distribution.

The undrawn (CM) and partially drawn samples (FC, FC-2) were investigated using thermogravimetric analysis (TGA, TGA-Q500, New Castle, DE, USA) to determine the remaining plasticizer content. First, the material was heated from 0 to 150 °C; then, it was held for 2 h to evaporate the GTA plasticizer until the residual weight reached a plateau. The weight loss was due to the plasticizer remaining after the manufacturing process.

Differential scanning calorimetry (DSC) was conducted using a Mettler Toledo DSC 822e (Columbus, OH, USA) in the temperature range of −20 to 200 °C at a heating rate of 10 °C/min to investigate the thermal behavior of the samples. The degree of crystallinity (%) of the samples was calculated using the following Equation (3) [23]:

(3) $$\mathrm{Crystallinity}\left(\%\right)=\frac{{\Delta H}_{m}-{\Delta H}_{c}}{{\Delta H}_{m}^{0}}\times \frac{100}{w}$$

where ΔH_m_ and ΔH_cc_ are the melting and cold crystallization enthalpies, respectively. ΔH^0^_m_ is the melting enthalpy of 100% crystalline PLA (93 J/g), and w is the weight fraction of PLA in the samples.

The mechanical properties of the undrawn and drawn nanocomposite films were tested using a Shimadzu AG-X universal tensile tester (Kyoto, Japan) with a 5 kN load cell; the length between the grips was 20 mm, and a crosshead speed of 2 mm/min was used. The tensile strength, elongation at break, tensile modulus, and toughness of the materials were determined from the data, and the toughness (work of fracture) was calculated as the area under the stress–strain curves. The results are the average of seven samples tested for each material.

A Nikon Eclipse LV100NPOL (Kanagawa, Japan) polarized optical microscope (POM) was used to analyze the effect of orientation on the microstructure of the nanocomposites and observe the birefringence behavior. The nanocomposite films were tested under cross-polarized conditions and polarized optical micrographs of the sample were recorded using a charge-coupled device camera.

The percentage transmittance of the un-oriented, pre-oriented, and oriented nanocomposites was investigated using a UV–Vis spectrophotometer (GENESYS, 10UV, Thermo-Scientific, Dreieich, Germany) at a constant wavelength of 220 nm, and three specimens of each sample were tested to calculate the average values.

The surface morphology was investigated using scanning electron microscopy (SEM, JEOL-IT 300, Tokyo, Japan). Tensile fracture surfaces and etched surfaces of undrawn and drawn nanocomposite films were examined. The samples were etched using sodium hydroxide and water (1:2 by volume) for 12 h. The surfaces of both the fractured and etched samples were coated with platinum (Leica EM ACE 220, Wetzlar, Germany) to avoid the charging effect.

Fourier transform infrared spectroscopy (FT-IR, VERTEX 80, Ettlingen, Germany) was used to study whether the orientation had any effect on the molecular interaction between the different components in the nanocomposites (PLA, GTA, and ChNCs). Scanning was performed in the spectral range 400–4000 cm^−1^ with a resolution of 128 cm^−1^.

## 3. Results

### 3.1. Crystal Structure and Orientation of the PLA–ChNC Nanocomposite Films

The structure of the crystalline phases and the orientation in the PLA–ChNC nanocomposite films were analyzed using X-ray diffraction.

**Figure 2:**
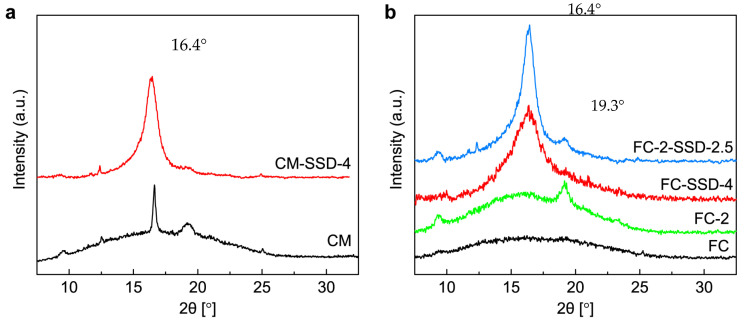
XRD patterns of the PLA–ChNC nanocomposite films with different degree of orientation. (**a**) isotropic compression molded film (CM) compared with solid-state drawn compression molded film (CM-SSD-4) and (**b**) film calendered film without drawing (FC) and calendered film with draw ratio 2 (FC-2) compared with solid-state drawn calendered films (FC-SSD-4 & FC-2-SSD-2.5).

As shown in Figure 2a, the undrawn CM sample showed a large broad peak associated with the amorphous phase of PLA, and small sharp peaks at 16.4° and 19.3° corresponding to the (110)/(200) and (203) planes of PLA crystallites, respectively [24]. This indicates that a small number of PLA crystals were generated during the CM process. Very small peaks at 9.4°, 12.5°, and 25.1° also appeared in this pattern that could be attributed to the 5 wt% of ChNCs in the nanocomposite; they were assigned to the (020), (021), and (130) crystal planes of chitin [25], respectively. However, the peak at 19.3° associated with the (110) chitin crystal plane overlaps with the (203) plane of PLA. In contrast, the FC sample shown in Figure 2b, only demonstrated a broad amorphous peak, and no crystalline peaks could be observed. After SSD, the CM-SSD-4 sample showed a much more intense but slightly broadened diffraction peak at 16.4° in Figure 2b, which can be attributed to the strain-induced crystallization behavior and decreased size of the PLA crystallites in the nanocomposite resulting from the drawing process [26]. The FC-SSD-4 sample with the same DR demonstrated a broader peak at the same 2θ angle, implying that the size of the PLA crystallites in this sample was smaller than that in CM-SSD-4, which was likely because the pre-orientation effect of the FC process resulted in more nucleation sites. Moreover, the diffraction peak at 19.3° was almost invisible in these two drawn samples, which was due to the limitation of the 1D XRD detector and confirms the orientation of the crystalline phases in the nanocomposite. In addition, the FC-2 pattern in Figure 2b had considerable stronger peaks at 9.4° and 19.3° compared to those of the FC film, probably because the further stretching in the pre-orientation process led to a different degree of orientation of the ChNCs in the nanocomposite. These features were maintained after the subsequent SSD process up to a DR of 2.5, and the WAXS pattern of FC-2-SSD-2.5 exhibited a narrower diffraction peak of the (110)/(200) PLA crystal planes compared to that of FC-SSD-4, revealing that larger and more complete PLA crystallites formed during the combined drawing approach.

**Figure 3:**
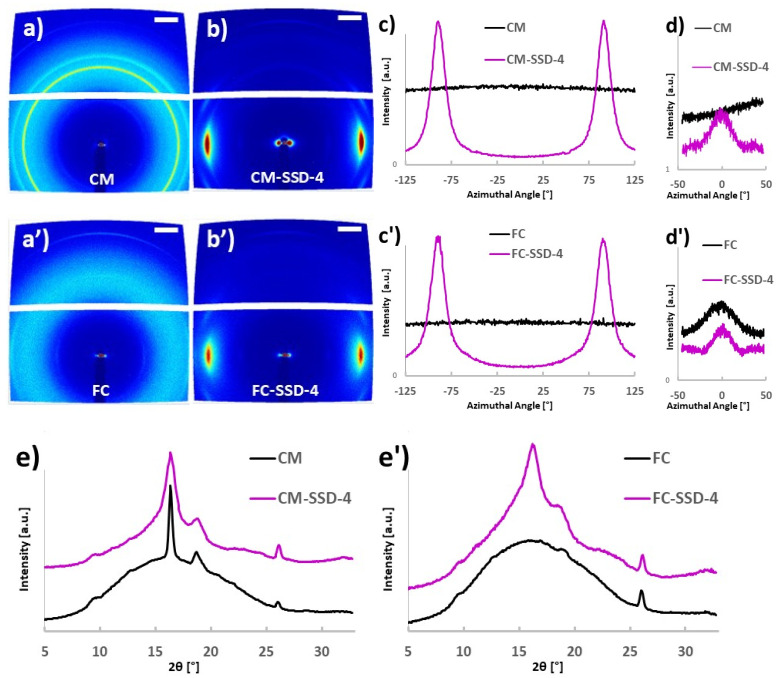
2D WAXS analysis of PLA and ChNC orientation in the nanocomposite films. (**a**,**a’**) 2D WAXS diffractograms of the undrawn nanocomposite films. The scale bar represents 2θ = 5°. (**b**,**b’**) 2D WAXS diffractograms of the drawn nanocomposite films. (**c**,**c’**) Azimuthal integration of the crystalline PLA scattering plane, 2θ = 16.4 ± 0.3°. (**d**,**d’**) Azimuthal integration of the ChNC scattering plane, 2θ = 26 ± 0.4°. (**e**,**e’**) Radial integration of the diffractograms. An offset has been added to the magenta-colored radial integration diffractograms to avoid overlapping curves.

Figure 3 shows 2D WAXS diffraction patterns and the related integration curves of the CM, FC, CM-SSD-4, and FC-SSD-4 films. Like the 1D WAXS results, both CM and FC exhibited broad diffraction peaks, indicating an amorphous structure. Relatively sharp crystal diffraction peaks, as well as broad diffraction peaks, were observed in WAXS diffractograms of the drawn nanocomposite films, indicating partial crystallization, see Figure 3e,e’. The main peak at 16.4° is characteristic for PLA and can be used to quantify the orientation of PLA crystalline phase in the nanocomposite films. By determining the maximum and baseline intensities in the intensity distribution as a function of the azimuthal angle of the 16.4° peak, the orientation index (*f_c_*) of PLA can be calculated according to Eq 2. If all PLA crystals are aligned in the same direction, *f_c_* = 1, and if they are randomly distributed, *f_c_* = 0. An isotropic diffraction ring was observed for the undrawn samples CM and FC, indicating that the PLA polymers were randomly oriented in the film plane, see Figure 3a,a’,c,c’. The orientation index for the CM-SSD-4 and FC-SSD-4 films was determined to be 0.9, indicating that the PLA crystals in these samples had a preferred orientation in the drawing direction, shown in Figure 3b,b’,c,c’. Interestingly, the peak at 26° which was assigned to the (130) plane of the ChNCs, is very distinct in the 2D WAXS results. Thus, it is possible to analyze the orientation of the ChNCs. For the undrawn CM sample, no clear maximum intensity can be observed in the azimuthal integration of the ChNC scattering plane, indicating that the ChNCs were randomly oriented in the film plane, shown Figure 3a,d. Surprisingly, the orientation index for ChNCs in the FC sample was 0.8, indicating that ChNCs were efficiently partially oriented in the nanocomposite film owing to the pre-orientation from the film calendering process. The ChNCs in both drawn samples had an orientation index value of 0.9, demonstrating that ChNCs reached a preferred orientation degree like that of the PLA crystalline phase in the film plane (Figure 3b,b’,d,d’).

The orientation of PLA and ChNC in the film plane and on the film edge (cross section) was also studied using 2D WAXS, as shown in Figure S2 in Supplementary Information. In the drawn films (CM-SSD-4 and FC-SSD-4), PLA and ChNCs were partially oriented in the drawing direction with a similar orientation index (*f_c_* = 0.9) in both the film plane and cross section. However, the undrawn CM and FC films exhibited different behaviors. The PLA crystals were randomly oriented in the plane of the CM and FC films, whereas in the CM and FC film cross sections, a minor part of the PLA crystals was partially oriented, as shown in Figure S2(A3 and C3). This can be attributed to the restriction effect caused by the relatively small film thickness. The ChNCs were randomly oriented in the CM film, both in the film plane and on the cross section, whereas in the FC film, the ChNCs were partially oriented, as shown in Figure S2(A4 and C4).

### 3.2. Effect of Orientation on Transparency

**Figure 4:**
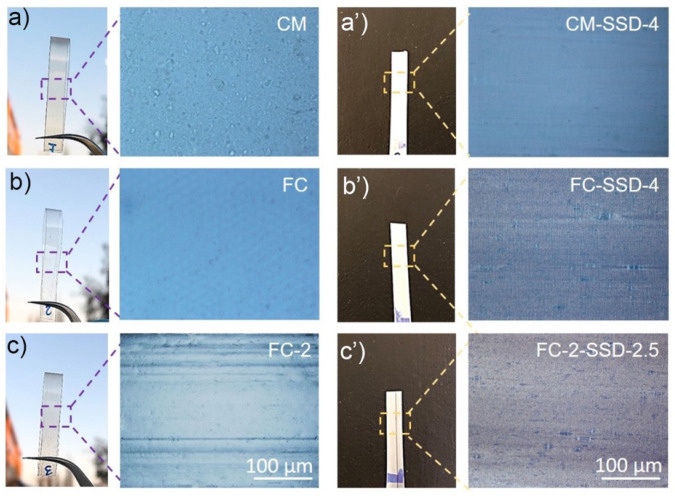
Visual appearance and optical micrographs of compression molded and film calendered nanocomposites before and after solid-state drawing. (**a**) compression molded film (CM); (**a’**) compression molded and solid-state drawn CM-SSD-4; (**b**) Film calendered film (FC); (**b’**) Film-calendered and solid-state drawn film FC-SSD-4; (**c**) Film calendered film with DR2 (FC-2); (**c’**) film-calendered and solid-state drawn film (FC-2-SSF-2.5). (All micrographs are taken with same magnification).

Photographs of the undrawn, calendered, and solid-state drawn nanocomposite films are shown in Figure 4. It can be clearly seen that the films were transparent before the SSD process Figure 4a–c some spots are visible in the compression molded film which might be associated to ChNCs Figure 4a. The films are transparent before the drawing and opaque/white after the orientation shown in Figure 4a’,b’,c’. It is also possible to see that the FC-2 film have horizontal marks in the draw direction and these marks are also visible after SSD (Figure 4c,c’).

### 3.3. Effect of Orientation on Thermal Properties

Before characterizing the properties of the nanocomposites, TGA was performed to study whether the CM and FC processes had different effects on the plasticizer content present in the obtained nanocomposite films. The TGA results are presented in the Supplementary Information in Table S1. These results show that the processing techniques used, and the additional drawing step had no significant effect on the remaining plasticizer content; approximately 7% weight loss due to GTA evaporation was detected in all the CM, FC, and FC-2 samples. This confirms that all samples maintained the same composition. Therefore, it did not influence the properties of the nanocomposites.

The thermal properties of the nanocomposites were studied using DSC. The results are shown in Table 3, and the DSC thermograms are provided in Figure S3 in Supplementary Information. Compared to that of the undrawn CM film, the FC film demonstrated a similar glass transition temperature (Tg) and melting temperature (Tm) and a slightly lower cold crystallization temperature (Tcc, 94 °C vs. 100 °C). The crystallinity of PLA in FC (13%) was slightly higher than that in CM (8%). This indicates that the FC process helped to pack the PLA chains in a more ordered state in the nanocomposite (i.e., the pre-orientation effect), which led to a small increase in crystallinity during the fast-cooling step in the manufacturing processes and a lower Tcc in the DSC heating run. For FC-2, the crystallinity drastically increased to 35% owing to the strain-induced crystallization, and the Tcc further decreased to 67 °C because the remaining ordered amorphous PLA chains could rapidly arrange to form a new crystalline phase with much lower thermal energy compared to that of those in the FC film. The Tg of FC-2 was also lower than that of FC, which could be attributed to the higher crystallinity leading to localization of the plasticizer in the amorphous region in the nanocomposite, as reported in our previous study [27]. For the solid-state drawn samples, the crystallinity increased to 46–53% because of the considerably higher degree of orientation caused by the higher draw ratios, and their Tg was not detected. Only very small Tcc peaks were observed, as shown in Figure S3, which were at almost the same temperature as that of FC-2. Like the undrawn samples, FC-SSD-4 had a slightly higher crystallinity than that of the CM-SSD-4, which was likely caused by the pre-orientation effect from the film calendering process, and FC-2-SSD-2.5 reached the highest crystallinity (53%). In addition, it is clear from Figure S3(A1) that FC-2 and all solid-state drawn samples showed asymmetric melting peaks, implying that large quantities of small PLA crystals were generated during the drawing process owing to the strain-induced crystallization effect.

### 3.4. Effect of Orientation on Mechanical Properties

The mechanical properties of the undrawn films (CM) compared with those of the oriented films with different orientation degrees are presented in Table 4. The results confirm that the orientation significantly affects all the mechanical properties, which is unusual behavior. An increase in stiffness and strength typically results in decreased elongation and toughness (work of fracture). In this study, FC increased the elongation at break from 3% to 24% and the work of fracture from 0.6 to 9 MJ/m^3^, while the stiffness and strength were only marginally improved.

When comparing nanocomposites oriented by SSD (CM-SSD-4) with pre-oriented nanocomposites (FC-SSD-4), it is seen that the strength was similar (135 and 140 MPa), but the modulus was higher for the pre-oriented ones, and the elongation at break and work of fracture increased substantially from 46% to 75% (63% increase) and 47 to 78 MJ/m^3^ (66% increase), respectively.

The highest mechanical properties were achieved for pre-oriented (FC-2-SSD-2.5) nanocomposites with the highest DR: a stiffness of 4 GPa, a strength of 170 MPa, an elongation at break of 75%, and a work of fracture of 96 MJ/m^3^. The percentage improvement compared to the undrawn composites was 74% for the stiffness, 360% for the strength, 2400% for the elongation, and 9500% for the work of fracture.

The marked improvement was due to the high orientation degree of the polymer chain as well as the strain-induced crystallization of PLA and the oriented ChNCs. The results indicate that the pre-orientation step not only oriented the polymer but also the ChNCs, which led to an overall increase in the mechanical properties.

The effect of the pre-orientation of FC-2 on the mechanical properties of the nanocomposites was investigated, and the results are shown in Table 4. As expected, pre-orientation of the nanocomposites affected the orientation and dramatically increased the mechanical properties compared to the undrawn materials. The mechanical properties of the PLA nanocomposites depend on several factors: (i) the adhesion between the PLA matrix and reinforcement phase, (ii) the aspect ratio of the reinforcement phase, (iii) the orientation and of the reinforcement and its ability to nucleate, (iv) the stress-transfer efficiency of the interface, and (v) the degree of crystallinity of the matrix [28,29,30].

The orientation of the undrawn film (CM) via SSD increased the overall mechanical properties. For example, the tensile modulus, tensile strength, elongation at break, and toughness of CM-SSD-4 increased by 13%, 266%, 1433%, and 7733%, respectively, compared to those of CM. This increase in the mechanical properties, particularly the tensile strength, elongation at break, and toughness, was due to the better stress-transfer efficiency that developed between the interface of plasticized PLA and ChNCs. As mentioned previously, a photograph of CM-SSD-4 showed opaque behavior, which further confirmed that the orientation of the PLA molecular chains caused by strain-induced crystallization resulted in a higher degree of crystallinity in the CM-SSD-4 nanocomposites. Noticeably, a small difference in standard deviations, particularly in the modulus and strength values, indicated that the ChNCs were well dispersed and distributed.

Furthermore, FC had a positive effect on the orientation and led to superior mechanical properties compared to those of CM-SSD-4, even at the same draw ratio (DR4). Consequently, the tensile modulus, tensile strength, and elongation at break of FC-SSD-4 increased by 50%, 4%, 63%, and 66%, respectively, with respect to those of CM-SSD-4. This increase in the tensile strength was due to the higher crystallinity and orientation obtained by FC. Further, the improvement in the elongation at break contributed to the toughness of the aligned PLA nanocomposites. Pre-orientation caused significant further improvement to the drawing ability and, hence, the mechanical properties of FC-2-SSD-2.5, for which a higher tensile strength and toughness were obtained as compared to those of FC-SSD-4. However, the tensile modulus and elongation at break remained the same. This could be due to crystallinity. As mentioned in the DSC results, there was no significant difference in the degree of crystallinity of FC-SSD-4 and FC-2-SSD-2.5 (51% for FC-SSD-4 and 53% for FC-2-SSD-2.5).

As expected, compared to the undrawn (CM) nanocomposites, highly drawn (FC-2-SSD-2.5) nanocomposites (i.e., highest total DR) showed the highest tensile modulus, strength, elongation at break, and toughness, which increased by 74%, 360%, 2400%, and 9500%, respectively. This was because of the better orientation of the ChNCs, and PLA molecular chains obtained by a combination of melt-state drawing and SSD, resulting in superior mechanical properties. It has been well documented in the literature that increasing the DR of the materials causes the mechanical properties to increase [14,15,16,17,18]. We can also compare the mechanical properties reached in this study with the previous study where TEC plasticized PLA and its nanocomposites with ChNCs were drawn with different draw ratios, reaching a maximum DR 3. The modulus of the SSD matrix (PLA-20TEC) was 0.76 GPa, strength 56 MPa and strain 92% compared with the nanocomposite PLA-20TEC-5ChNC, in which the modulus was 1.72 GPa, strength 72 MPa, and strain 59% [18]. The previous study clearly showed the influence of the ChNCs. It can be concluded that by combining these two techniques, the overall mechanical properties of the nanocomposites have massively improved as compared to the previous studies reported in the literature.

### 3.5. Effect of Orientation on Morphology

**Figure 5:**
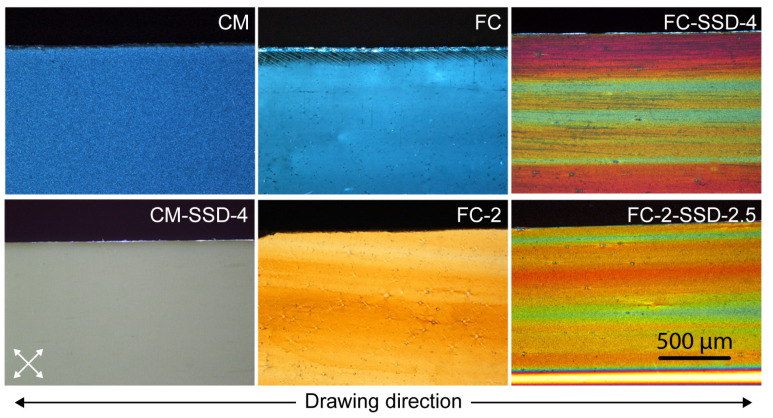
Polarized optical micrographs of the undrawn and drawn nanocomposite films. Directions of the cross polarizers are shown on the bottom left corner, and the drawing direction of the films is shown on the bottom. The FC-SSD-5 oriented film showed the brightest and most colorful birefringence because of the orientation of the PLA.

To better understand and visualize how different processing routes and draw ratios induced the orientation in the nanocomposites, polarized optical micrographs of undrawn and drawn films were compared and these are shown in the Figure 5. Generally, orientation of the PLA results in strain-induced birefringence. The color produced during drawing depends on the degree of orientation of the molecular chains of the polymer. In this study, undrawn samples showed either no color or a single color (shown in Figure 5 CM, FC). The CM-SSD-4 nanocomposite exhibited a gray shade due to strain hardening effect, which resulted from larger spherulites that developed during stretching that prohibited light from passing through the samples as seen in the CM-SSD-4 on bottom row in Figure 5. However, the pre-oriented film-calendered materials, at the same DR 4, demonstrated very bright birefringence, which were induced by the synergistic effect of ChNCs, and orientation PLA achieved by FC combined with SSD. Additionally, the drawn samples (FC-2-SSD-2.5) exhibited a very homogenous orientation, which can be attributed to the good dispersion and distribution of the ChNCs in the PLA.

**Figure 6:**
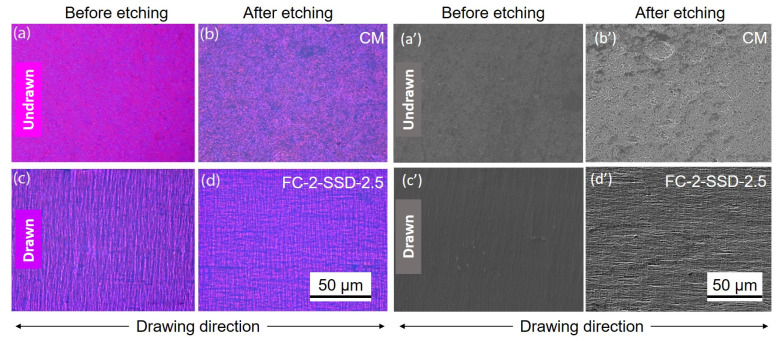
(**a**–**d**) POM and (**a’**–**d’**) SEM micrographs of the CM and FC-2-SSD-2.5 nanocomposite films before and after etching. The drawn nanocomposite shows orientation lines before etching and lines perpendicular to the drawing direction after etching, which confirm the shish-kebab morphology.

Furthermore, surface morphologies of the compression molded (CM) and film calendered solid state drawn nanocomposites were examined. The film surfaces were etched to better obtain the surface morphology, the materials studied in polarized microscopy and electron microscopy are shown in Figure 6.

As seen in the Figure 6a,b,a’,b’, the CM shows no orientation lines (patterns) on the surface, either before or after etching. In contrast, the highest drawn nanocomposite (FC-2-SSD-2.5) exhibited a highly aligned morphology, seen in Figure 6c,d,c’,d’. Noticeably, in the FC-2-SSD-2.5 films, a significant crazing effect was also visible perpendicular to the drawing direction. In addition, after etching, a cross-patterned morphology was observed, which is attributed to etching of the amorphous regions of the polymer. It is worth mentioning that the development of this cross-patterned structure in the drawn nanocomposites is due to the known shish-kebab morphology, which forms by folding of polymer chains [14,15].

FT-IR was also conducted to understand the influence of orientation on the structure of the nanocomposites. The spectra of CM and FC-2-SSD-2.5 are presented in Figure S4 in Supplementary Information. The drawn nanocomposites exhibited a reduced intensity compared to that of the undrawn nanocomposites. The main difference was observed in the fingerprint region, particularly in the range 1200–1000 cm^−1^. The undrawn (CM) nanocomposites showed peaks at 1082 and 1180 cm^−1^, which shifted to higher wavenumbers in the drawn nanocomposites (1089 and 1184 cm^−1^, respectively). This can be attributed to the intermolecular interactions developed by PLA with ChNCs and GTA.

It was shown that the orientation of the structure greatly influenced the properties of the PLA nanocomposites. A schematic of the ordering of the molecular chains during the orientation is shown in Figure S5 in Supplementary Information. Semicrystalline polymers exhibit two regions: amorphous and crystalline. Generally, the amorphous region plays an important role during stretching or stress. In this study, during melt-state drawing, the PLA chain must have folded together and formed some spherulites, as depicted in Figure S5. SSD was performed at 60 °C; therefore, during stretching, some tie molecules must have developed in the PLA nanocomposites, particularly in the amorphous region. Owing to the formation of tie molecules in the amorphous region of the polymer, the mechanical properties of the drawn nanocomposites increased, particularly the toughness and strength. These tie molecules can attach to the crystalline lamellae and facilitate the flexibility of the nanocomposites. The reason for the formation of the tie molecules could be the presence of –NH_2_–CO– groups in the molecular structure of chitin, which can form hydrogen bonds with the polymer (PLA) and plasticizer (GTA).

## 4. Conclusions

Oriented PLA–GTA–ChNC nanocomposite films were produced by combining FC and SSD. The two-step orientation procedure influenced the overall properties of the nanocomposites. Furthermore, the pre-orientation of the polymer chains achieved by FC enhanced the flexibility of the polymeric chains and ChNCs, resulting in improved properties.

The effect of orientation on the thermal properties, mechanical properties, surface morphology, and structural behavior of the nanocomposite was studied. The results confirmed that pre-orienting the nanocomposites with calendering had a pronounced effect on the thermal properties. This was due to the orientation of the ChNCs and PLA molecular chains, as well as strain-induced crystallization, which together led to improved mechanical properties.

Multifold increments were observed in the tensile modulus (2-fold), tensile strength (5-fold), elongation at break (25-fold), and toughness (96-fold) of the nanocomposites with the highest orientation degree.

Furthermore, the surfaces of the un-oriented and oriented nanocomposite films were observed using polarized microscopy. The oriented films exhibited more homogenous colors owing to the strain-induced birefringence of the PLA developed by drawing. It was clear that highly drawn films showed more colors owing to the alignments of the molecular chains.

The XRD study showed a reduction in the crystallite size due to the orientation of the nanocomposite films. The results demonstrate that increasing the orientation of the nanocomposite structure is an extremely efficient way to produce films with high strength and toughness, which is a potential path for the development of sustainable materials for packaging applications.

## Figures and Tables

**Table 1:** Processing parameters used for calendering to produce pre-oriented nanocomposite films.

Materials	Temperature (°C)			Speed (mm/min)		
	Extruder	Die	Stack Rolls	Stack Rolls	Wind-Up Rolls	DR
FC *	190	200	60	18	18	--
FC-2 **	190	200	60	18	36	2

* FC indicates film calendering, and ** FC-2 denotes film-calendered samples drawn at DR = 2.

**Table 2:** Coding of the nanocomposites based on the different processing methods and obtained draw ratio (DR).

Codes	Processing	Drawing	Total DR
CM	Compression molding	-	-
CM-SSD-4	Compression molding	Solid-state drawing	4
FC	Film calendering	-	-
FC-2	Film calendering	Calendering	2
FC-SSD-4	Film calendering	Solid-state drawing	4
FC-2-SSD-2.5	Film calendering	Calendering and solid-state drawing	5

**Table 3:** Thermal properties determined from the first heating scans of undrawn, pre-drawn and drawn nanocomposite films drawn at 60 °C.

Materials	T_g_ (°C)	T_cc_ (°C)	T_m_ (°C)	Crystallinity (%)
CM	55	100	169	8
CM-SSD-4	---	65	170	46
FC	54	94	170	13
FC-2	48	67	171	35
FC-SSD-4	---	66	173	51
FC-2-SSD-2.5	---	63	171	53

**Table 4:** Mechanical properties of the undrawn (CM), calendered pre-oriented films (FC), and solid-state drawn nanocomposites (SSD) with three different draw ratios.

Materials	Tensile Modulus (GPa)	Tensile Strength (MPa)	Elongation at Break (%)	Work of Fracture (MJ/m^3^)
CM	2.3 (±0.1)	37 (±3)	3 (±1)	1 (±0)
CM-SSD-4	2.6 (±0.1)	135 (±3)	46 (±2)	47 (±3)
FC	2.5 (±0.2)	41 (±2)	24 (±6)	9 (±1)
FC-2	2.9 (±0.2)	58 (±3)	143 (±5)	59 (±2)
FC-SSD-4	3.9 (±0.2)	140 (±7)	75 (±9)	78 (±9)
FC-2-SSD-2.5	4.0 (±0.2)	170 (±10)	75 (±9)	96 (±12)

## Data Availability

Data is available by request.

## Supplementary Materials

The following are available online at https://www.mdpi.com/article/10.3390/nano11123308/s1, Figure S1: Schematic representation of (a) melt-state drawing and (b) SSD of PLA nanocomposite films conducted on extrusion and film calendaring and uniaxial tensile tester with a temperature chamber, respectively. Figure S2: 2D WAXS analysis of PLA and ChNC orientation in the nanocomposite films. Column (1) 2D WAXS diffractograms of the X-ray beam through the film plane. The white scale bar represents 2θ = 5°. Column (2) 2D WAXS diffractograms of the X-ray beam through the film edge (cross-section). Column (3) Azimuthal integration of the crystalline PLA scattering plane, 2θ = 16.4 ± 0.3°. Column (4) Azimuthal integration of the ChNC scattering plane, 2θ = 26 ± 0.4°. (Row A) Undrawn compression molded nanocomposite films. (Row B) Drawn compression molded nanocomposite films. (Row C) Film-calendared nanocomposite films. (Row D) Drawn calendared nanocomposite films. DSC thermograms of the nanocomposite films taken from the first heating run. Figure S3: DSC thermograms of the nanocomposite films taken from the first heating run. Figure S4: ATR-FTIR of undrawn CM and drawn FC-2-SSD-2.5 nanocomposite films. Figure print region of the spectra showing that bands of drawn nanocomposite are shifting towards the higher wave number. Figure S5: Schematics representing the ordering of the polymer chains during the orientation of the nanocomposites (a) arrangement of the polymer chains to form the ordered lamella (b) shows the amorphous and crystalline part of the PLA (c) formation of tie molecules in the amorphous part of PLA due to the orientation. Table S1: Thermal properties obtained from TGA data of the nanocomposite films from compression molding and film calendaring to measure the remaining plasticizer content in the films.
