# Supplementary material for: Orientation of Polylactic Acid–Chitin Nanocomposite Films via Combined Calendering and Uniaxial Drawing: Effect on Structure, Mechanical, and Thermal Properties
Source: Nanomaterials (Basel). 2021 Dec 6;11(12):3308. doi: 10.3390/nano11123308 (PMC8706151; doi:10.3390/nano11123308)
Supplement: Supplementary file 1 [file nanomaterials-11-03308-s001.zip › nanomaterials-1472882-supplementary.pdf]

nanomaterials

Supplementary Materials

Orientation of Polylactic Acid–Chitin Nanocomposite Films via Combined Calendering and Uniaxial Drawing: Effect on Structure, Mechanical, and Thermal Properties

Shikha Singh [1,2], Mitul Kumar Patel [1], Shiyu Geng [1,] Anita Teleman [3], Natalia Herrera [1], Daniel Schwendemann [1,4], Maria Lluisa Maspoch [2] and Kristiina Oksman [1,5,6,*]

[1] Division of Materials Science, Department of Engineering Sciences and Mathematics, Luleå University of Technology, SE-971 87 Luleå, Sweden; sikha.msc@gmail.com (S.S.); mitul.kumar.patel@ltu.se (M.K.P.); Shiyu.geng@ltu.se (S.G.); natalia.herrera-vargas@storaenso.com (N.H.); kristiina.oksman@ltu.se (K.O.)
[2] Centre Català del Plàstic (CCP)-Universitat Politècnica de Catalunya Barcelona Tech (EEBE-UPC)-ePLASCOM, 08019 Barcelona, Spain; maria.lluisa.maspoch@upc.edu
[3] RISE (Research Institutes of Sweden), SE-114 28 Stockholm, Sweden; anita.teleman@ri.se
[4] OST Eastern Switzerland University of Applied Sciences, CH-8640 Rapperswil, Switzerland; daniel.schwendemann@ost.ch
[5] Mechanical & Industrial Engineering, University of Toronto, Toronto, ON M5S 3BS, Canada
[6] Wallenberg Wood Science Center (WWSC), Luleå University of Technology, SE-971 87 Luleå, Sweden
* Correspondence: Kristiina.oksman@ltu.se

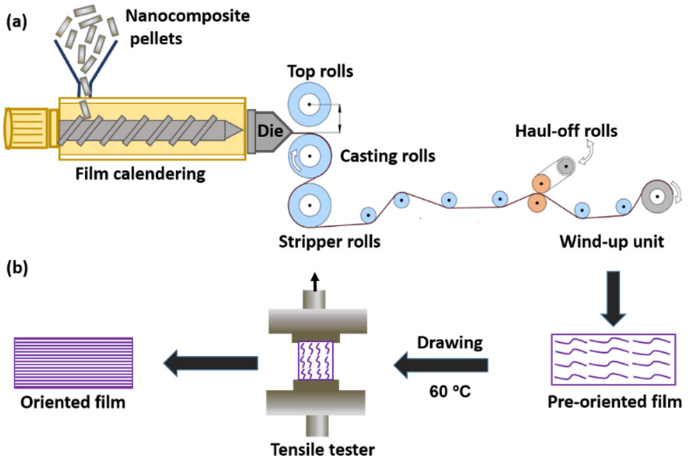

Figure S1. Schematic representation of (a) melt-state drawing and (b) SSD of PLA nanocomposite films conducted on extrusion and film calendaring and uniaxial tensile tester with a temperature chamber, respectively.

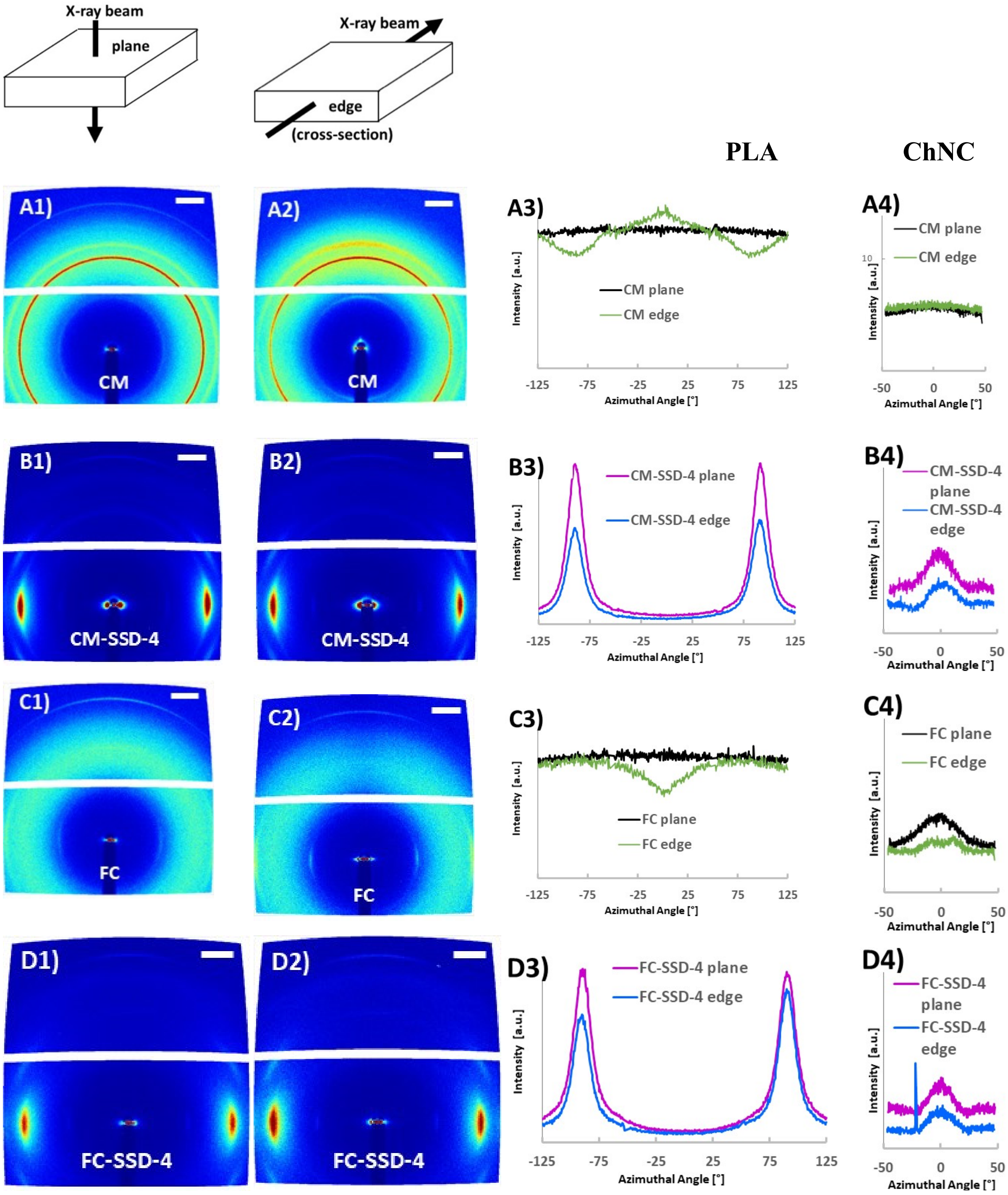

Figure S2. 2D WAXS analysis of PLA and ChNC orientation in the nanocomposite films. Column 1) 2D WAXS diffractograms of the X-ray beam through the film plane. The white scale bar represents 2θ = 5°. Column 2) 2D WAXS diffractograms of the X-ray beam through the film edge (cross-section). Column 3) Azimuthal integration of the crystalline PLA scattering plane, 2θ = 16.4±0.3°. Column 4) Azimuthal integration of the ChNC scattering plane, 2θ = 26±0.4°. Row A) Undrawn compression molded nanocomposite films. Row B) Drawn compression molded nanocomposite films. Row C) Film-calendared nanocomposite films. Row D) Drawn calendared nanocomposite films.

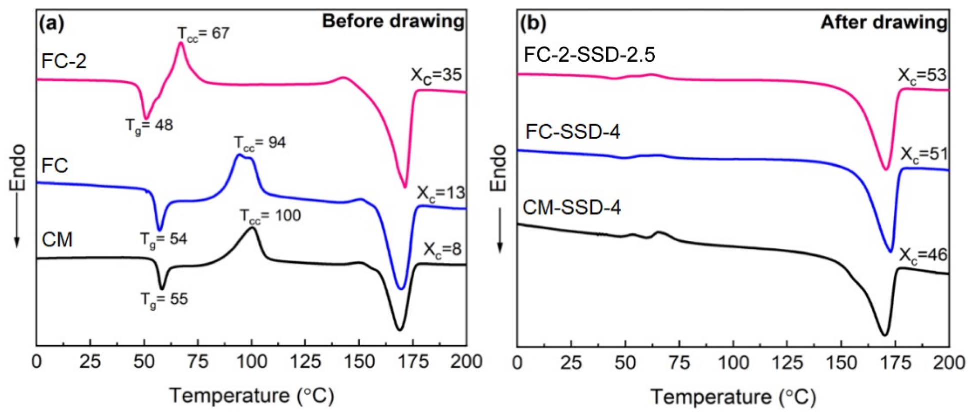

Figure S3. DSC thermograms of the nanocomposite films taken from the first heating run.

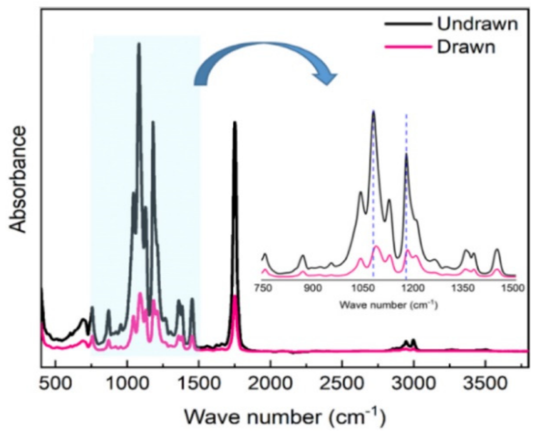

Figure S4. ATR-FTIR of undrawn CM and drawn FC-2-SSD-2.5 nanocomposite films. Figure print region of the spectra showing that bands of drawn nanocomposite are shifting towards higher wave numbers.

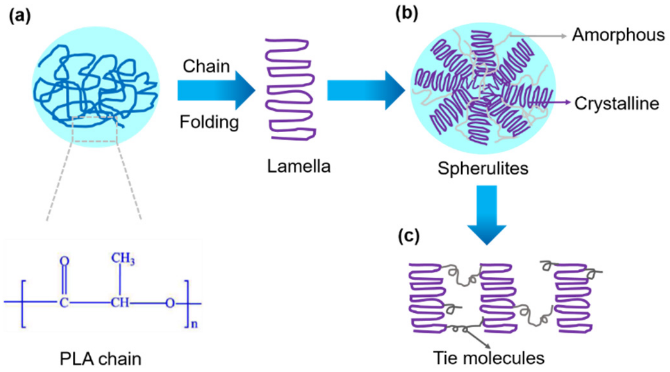

Figure S5. Schematics representing the ordering of the polymer chains during the orientation of the nanocomposites (a) arrangement of the polymer chains to form the ordered lamella (b) shows the amorphous and crystalline part of the PLA (c) formation of tie molecules in the amorphous part of PLA due to the orientation.

Table S1. Thermal properties obtained from TGA data of the nanocomposite films from compression molding and film calendaring to measure the remaining plasticizer content in the films.

Materials	Initial Value	Final Value	Lost plasticizer (GTA) (%)
CM	100	93.3	6.7
FC	100	93.2	6.7
FC-2	100	93.4	6.6
